# Preoperative robotic radiosurgery for early breast cancer: Results of the phase II ROCK trial (NCT03520894)

**DOI:** 10.1016/j.ctro.2022.09.004

**Published:** 2022-09-22

**Authors:** Icro Meattini, Giulio Francolini, Vanessa Di Cataldo, Luca Visani, Carlotta Becherini, Erika Scoccimarro, Viola Salvestrini, Chiara Bellini, Laura Masi, Raffaela Doro, Federica Di Naro, Mauro Loi, Giulia Salvatore, Gabriele Simontacchi, Daniela Greto, Marco Bernini, Jacopo Nori, Lorenzo Orzalesi, Simonetta Bianchi, Monica Mangoni, Lorenzo Livi

**Affiliations:** a Department of Experimental and Clinical Biomedical Sciences “M. Serio”, University of Florence, Florence, Italy; b Radiation Oncology Unit, Oncology Department, Azienda Ospedaliero Universitaria Careggi, Florence, Italy; c CyberKnife Center, Istituto Fiorentino di Cura e Assistenza (IFCA), Florence, Italy; d Diagnostic Senology Unit, Azienda Ospedaliero Universitaria Careggi, Florence, Italy; e Breast Surgery Unit, Azienda Ospedaliero Universitaria Careggi, Florence, Italy; f Division of Pathological Anatomy, Department of Health Sciences, University of Florence, Florence, Italy

**Keywords:** Breast cancer, Preoperative radiotherapy, Robotic radiosurgery, Partial breast irradiation

## Abstract

**Background and purpose:**

Preoperative partial breast irradiation (PBI) has got the advantage of treating a well-defined target. We report the results of the phase II ROCK trial (NCT03520894), enrolling early breast cancer (BC) patients treated with preoperative robotic radiosurgery (prRS), in terms of acute and early late toxicity, disease control, and cosmesis.

**Material and methods:**

The study recruited between 2018 and 2021 at our Radiation Oncology Unit. Eligible patients were 50 + years old BC, hormonal receptors positive/human epidermal growth factor receptor 2 negative (HR+/HER2-), sized up to 25 mm. The study aimed to prospectively assess the toxicity and feasibility of a robotic single 21 Gy-fraction prRS in preoperative setting.

**Results:**

A total of 70 patients were recruited and 22 patients were successfully treated with pRS. Overall, three G1 adverse events (13.6 %) were recorded within 7 days from prRS. Three events (13.6 %) were recorded between 7 and 30 days, one G2 breast oedema and two G1 breast pain. No acute toxicity greater than G2 was recorded. Five patients experienced early late G1 toxicity. One patient reported G2 breast induration. No early late toxicity greater than G2 was observed. At a median follow up of 18 months (range 6–29.8), cosmetic results were scored excellent/good and fair in 14 and 5 patients, respectively, while 3 patients experienced a poor cosmetic outcome.

**Conclusions:**

ROCK trial showed that a single 21 Gy dose prRS represents a feasible technique for selected patients affected by early BC, showing an acceptable preliminary toxicity profile.

## Highlights

• Preoperative partial breast irradiation treats well-defined target.
• Stereotactic body radiation therapy have been routinely implemented in clinical practice.
• No acute toxicity greater than grade 2 was recorded.
• A single 21 Gy dose preoperative robotic radiosurgery represents a feasible technique.

## Introduction

Breast-conserving surgery (BCS) followed by postoperative radiation therapy (RT) still represents the standard of care for most of the early breast cancer (BC) patients, since this strategy allowed a significant reduction of mastectomy rates with functional, cosmetic, and psychological benefit [1], [2]. Hypofractionated schedules in maximum 15 fractions are currently accepted as the gold standard for external beam whole and partial breast irradiation (PBI) [3]. Moreover, accelerated external beam PBI, intraoperative irradiation, and brachytherapy for selected early BC patients allowed a shorter overall treatment duration and an improved patient compliance as compared to the old-fashioned RT schedules [4], [5], [6], [7], [8].

One of the main concerns in the postoperative setting is the uncertainty about surgical bed definition, prompting to increase treatment volume to include all violated areas within the target. For this reason, preoperative RT, due to the advantage of treating a well-defined volume, has been gaining attention in many clinical scenarios, including BC [9]. It avoids local treatment delay and may allow potential tumour downstaging with increased rates of BCS and theoretically improved cosmetic outcomes [10].

Moreover, it is assumed that most relapses occur within the primary tumour site, regardless of RT use and surgical margins status [11], [12]. Thus, it is supposed that local recurrence is driven by biological mechanisms of radio-resistance, rather than geographical miss. Higher dose per fraction may overcome repair mechanisms allowing tumoral cells to escape from conventional ionizing radiation damage. Several observations suggested that BC is sensitive to hypofractionation [13]. High dose gradient techniques, such as stereotactic body radiation therapy (SBRT), have been routinely implemented in clinical practice thanks to their widespread availability, and are currently used as a curative treatment in several diseases (i.e., non-small cell lung cancer, prostate cancer) [14], [15]. Several studies assessed the feasibility of PBI using multiple techniques in the preoperative setting followed by standard BCS [16], [17], [18], [19], [20]. Cyberknife® (Accuray Incorporated, Sunnyvale, CA, USA) is a frameless robotic stereotactic radiosurgery system, providing continuous motion tracking during respiratory movement. This peculiarity, together with the use of multiple non-coplanar fields, allows to improve non target tissue sparing. For these reasons, Cyberknife® emerged as a potential alternative to standard PBI techniques, and preliminary experiences reported excellent cosmetic outcomes [21].

Aiming to exploit these technical advantages in an emerging framework, we designed a phase II trial (ROCK trial – NCT03520894) enrolling early BC patients treated with preoperative robotic radiosurgery (prRS). Here, we report the results of the study in terms of acute and early late toxicity, disease control, and cosmetic outcome.

## Material and methods

### Study population

This trial recruited between August 2018 and September 2021 at the Radiation Oncology Unit of the University of Florence (Florence, Italy). Eligible patients were women aged 50 years or older, with histologically proven invasive early BC, hormonal receptors positive/human epidermal growth factor receptor 2 negative (HR+/HER2-) disease, tumour size up to 25 mm suitable for BCS. Exclusion criteria were clinical node positive disease, multiple foci tumours and, to limit the risk of RT-related skin toxicity, patients with breast lesion limiting within 5 mm from the skin surface. At time of recruitment a diagnosis of invasive breast carcinoma was provided. Waiting for the final specimen report on biology (HR status, HER2 status, Ki67 proliferative index) patients were required to sign the informed consent in order to receive fiducials markers placement together with preoperative tumour localisation markers in a one-time procedure.

### Endpoints

The study aimed to prospectively assess the toxicity and feasibility of a single Cyberknife® (Accuray Incorporated, Sunnyvale, CA, USA) 21 Gy-fraction prRS in early BC preoperative setting, and to identify predictive factors for outcomes based on biological and clinical features. The primary endpoint was the acute skin toxicity (within 6 months from prRS) according to the toxicity criteria of the Radiation Therapy Oncology Group (RTOG) and the European Organization for Research and Treatment of Cancer (EORTC) scale [22]. Assuming an acute skin toxicity rate (any grade) of 19.9 % based on our previous experience on accelerated PBI [7], a minimum sample size of 22 patients would be needed to estimate this endpoint with a 14 % precision margin and a 90 % confidence interval.

Secondary endpoint related to treatment toxicity was the rate of non-skin acute toxicity. Rate of postoperative complications related to breast surgery (seroma, infection, hematoma, wound dehiscence, persistent postsurgical pain, and venous thromboembolism) was collected and reported. Cosmetic outcomes were prospectively scored every-six months using the BCCT.core software [23]. Secondary endpoints related to treatment efficacy were rate of pathological complete response (pCR) according to Chevallier score [24]. Explorative translational objectives of the trial were the evaluation of biomarkers associated to pCR and will be the object of a separate report.

The study was conducted according to the Declaration of Helsinki and the Guidelines for Good Clinical Practice. All patients provided full written informed consent. Trial approval was provided by the local Ethical Committee Area Vasta Centro (CEAVC, approval number 10936). This trial is registered on ClinicalTrials.gov (Identifier NCT03520894).

### Treatments

Cyberknife® is a high-precision robotic system used for SBRT delivery; thanks to an elevated number of non-coplanar beams, it allows greater conformity index with significant dosimetric advantage when compared to standard treatment. All patients eligible for the study according to inclusion criteria underwent fiducial markers’ introduction in *peri*/intralesional position (range 3–5 markers). Contrast enhanced planning computed tomography (CT) in supine position, with 1.25 mm slice thickness, was performed at least one week after fiducial markers positioning. Magnetic resonance imaging (MRI) was performed in treatment (supine) position and co-registered with planning CT to identify contrast enhancing tumour. Planning CT images were uploaded on Precision Treatment Planning System (Accuray Incorporated, Sunnyvale, CA, USA).

Gross tumour volume (GTV) was delineated on contrast enhanced planning CT, taking in account co-registered MRI and available clinical information. Clinical target volume (CTV) was obtained adding a 15 mm expansion to GTV [25]; thoracic wall and pectoral muscles were excluded from CTV, limitation at 5 mm from the skin surface was applied. An additional 3 mm margin excluding the first 5 mm of subcutaneous tissue was added to generate the planning target volume (PTV). Contoured organs at risk (OARs) were bilateral breasts, skin (defined as a 3-mm layer from the external body surface), thoracic wall, lungs, heart, thyroid, and spinal cord. A single fraction of 21 Gy to a minimum prescription isodose of 95 % was prescribed to PTV (corresponding to a maximum dose within PTV <27.3 Gy). A single 21 Gy fraction was chosen based on prior evidence for efficacy and limited toxicity evidenced within prospective trials evaluating intraoperative irradiation [5]. According to linear quadratic model, a 21 Gy single fraction corresponds to a biologically effective dose of 65 Gy using an alpha/beta ratio of 10 Gy. However, a 21 Gy single fraction treatment would correspond to a BED of 131 Gy assuming for BC a lower alpha/beta ratio of 4 Gy [26]. Dose constraints used for OARs derived from NSABP B39/RTOG 0413 trial, after adaptations to consider the single fraction schedule (Supplemental Table 1) [18], [27].

Dose to target conformity was evaluated in terms of the new conformity index (CI), calculated by the Cyberknife® as:

$$\mathrm{nCI}\;=\;\frac{\mathrm{PTV}\;\times \;PIV}{\mathrm{TI}V^{2}}$$

where PIV is the prescription isodose volume and TIV is the tumour volume covered by the prescription isodose; this index is the inverse of the Paddick CI [28].

**Fig. 1:**
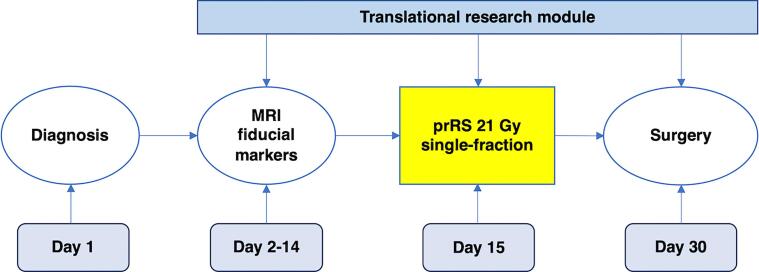
Study overview: a step-by-step overview of ROCK trial. Abbreviations: MRI, magnetic resonance imaging; prRS, preoperative radiosurgery.

Patients received BCS two weeks after prRS, keeping unaltered our local waiting time of receiving surgery at latest four weeks after BC diagnostic biopsy (Fig. 1). Adjuvant chemotherapy and/or endocrine treatment were prescribed as clinically indicated after final pathology results and postoperative BC multidisciplinary board meeting. re-excision was recommended for all patients reporting inadequate final surgical margins (namely, close <1 mm or positive margins). Postoperative whole breast irradiation was delivered if unsuitable features for accelerated PBI as per Groupe Europeen de Curietherapie-European Society for Therapeutic Radiology and Oncology (GEC-ESTRO) BC working group were detected at final specimen evaluation [29].

### Follow-up

After completion of prRS, we followed-up all patients after one month and every 6 months, thereafter. Clinical examination was performed at each follow-up visit; mammography was planned annually. Other diagnostic examinations were performed only in case of suspect symptoms. Baseline heart ultrasound and spirometry were performed before prRS and recommended yearly thereafter. RT treatment toxicity was assessed using the acute radiation morbidity scoring scheme from the RTOG and the EORTC [22]. A translational research module was conducted to identify correlations between radio-genomic, immunological, and biochemical biomarkers potentially predictive of treatment response and toxicity; main results are not mature at present analysis and will be reported at a later stage. Translational research module methods are summarized in the Supplemental Table 2.

## Results

### Patient characteristics and treatment

**Fig. 2:**
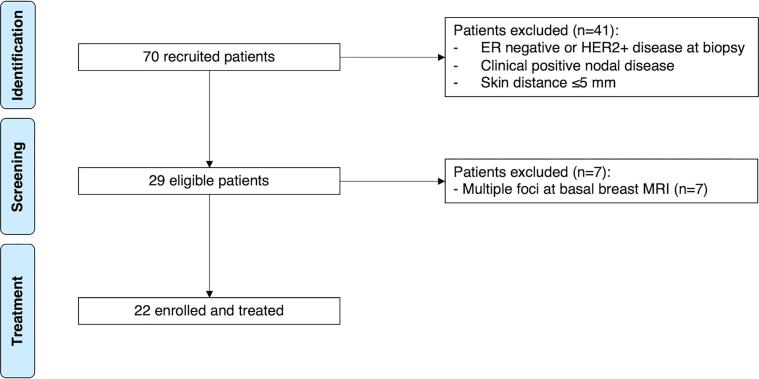
Phase II ROCK trial (NCT03520894) flow diagram.

From August 2018 to September 2021, a total of 70 patients were recruited and enrolled in this trial; of those, 41 were excluded due to tumour biology exclusion criteria and 7 due to multiple foci breast disease evidenced at baseline MRI. Biology features were provided one week after recruitment; a not negligible rate of patients eligible at diagnostic and clinical assessments were excluded after final immunohistochemistry tumour biopsy biology report. Therefore, 22 patients were successfully treated with pRS (Fig. 2).

Main population characteristics at baseline are summarized in Table 1. Median age at diagnosis was 68 years (range 50–86) and median tumour size was 14 mm (range 7.5–25). The median follow-up of the series was 18 months (range 6–29.8). Required prRS target dosimetric parameters were met in all patients, as well as normal tissue constraints (Table 2). Mean heart dose was 0.63 Gy (median 0.66 Gy, range 0.22–1.02 Gy), mean ipsilateral lung dose 0.91 Gy (median 0.89 Gy, range 0.16–1.51 Gy), mean ipsilateral breast dose 6.79 Gy (median 7.22 Gy, range 3.87–9.94 Gy).

**Table 1:** Baseline and postoperative patients’ characteristics (n=22).

Feature	Patients (n, %)
Median age at baseline, years (range)	67.5 (50-86)
Median Tumour size, mm (range)	13 (7.5-25)

Primary tumour location	
Right Side	15 (68.2)
Left Side	7 (31.8)

Involved Breast Quadrant	
Upper Outer	11 (50)
Upper Central	3 (13.6)
Upper Inner	3 (13.6)
Lower Central	2 (9.1)
Lower Inner	2 (9.1)
Subareolar	1 (4.6)

Ki67 proliferative index	
<20%	13 (59.1)
≥20%	9 (40.1)

ER status	
≥20%	22 (100)
<20%	0 (0)

PgR status	
≥20%	20 (90.9)
<20%	2 (9.1)

Pathological T stage	
ypT0	2 (9.1)
ypT1	19 (86.4)
ypT2	1 (4.5)

Pathological N stage	
ypN0	19 (86.4)
ypN1	3 (13.6)

Postoperative treatments	
Exclusive endocrine therapy	18 (81.8)
Endocrine therapy and chemotherapy	3 (13.6)
Whole breast irradiation	2 (9.1)
None	1 (4.6)

Abbreviations. ER, oestrogen receptor; PgR, progesterone receptor.

**Table 2:** Dosimetry assessment of treated patients (n=22).

	Median	Range
GTV (cc)	8.15	1.53–24.92
CTV (cc)	78.98	27.02–142.03
PTV (cc)	100.63	37.97–181.35
IDL (%)	78.6	77.4–83.6
PTV Coverage (%)	95.11	95.07–96.98
Dmax PTV (cGy)	2673.5	2512–2727
Dmean PTV (cGy)	2331.5	2268–2385
Dmin PTV (cGy)	1587.5	1388–1747
CI	1.135	1.08–1.27
Ipsilateral Breast V10.5Gy (%)	26.15	10.8–38.7
Ipsilateral Breast V21Gy (%)	12.45	4.6–18.3
Contralateral Breast Dmax (cGy)	75.5	8–175
Ipsilateral Lung V7Gy (cc)	2.7	0–16.82
Contralateral Lung Dmax (cGy)	107.5	56–210
Heart V3Gy (cc)	0.005	0–16.82
Chest Wall V10Gy (cc)	8.955	0–12.39
Chest Wall Dmax(cGy)	2049.5	308–2466
Skin Dmax (cGy)	1737	1882–1384
Skin V10Gy (cc)	8.45	4.41–12.04
Skin D10cc(cGy)	946.5	721–1051
Skin D1cc (cGy)	1382	1289–1484

Abbreviations. GTV, gross tumour volume; CTV, clinical target volume; PTV, planning target volume; IDL, isodose line; CI, Conformity Index.

### Treatment-related toxicity and complications

All patients underwent planned surgery after a median time of 29 days from biopsy (range 14–50), without any delay or complication. Overall, three grade 1 (G1) adverse events were recorded within seven days from prRS (one erythema, two breast pain). Three events were recorded between 7 and 30 days from prRS, one grade 2 (G2) breast oedema and two G1 breast pain. No acute toxicity greater than G2 was recorded.

**Fig. 3:**
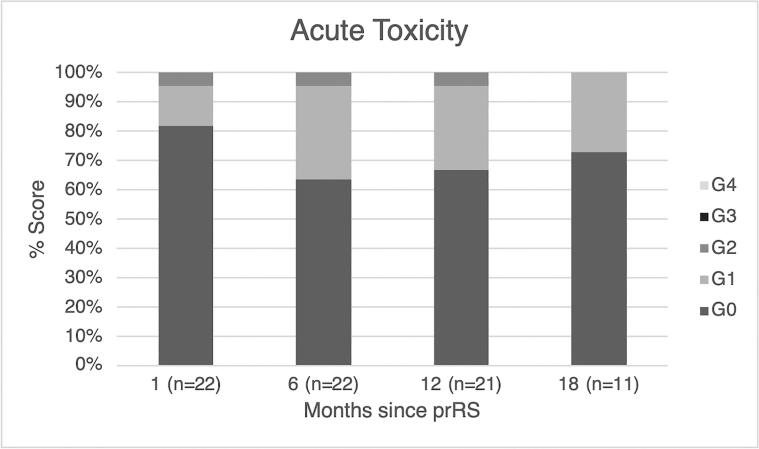
Acute toxicity at different time-points.

Five patients experienced G1 toxicity (one breast pain, four breast induration) after 30 days from prRS. One patient reported G2 breast induration after 30 days from prRS. No toxicity greater than G2 was observed. No postoperative complications related to breast surgery was reported. The grade of acute toxicity at different time-points are reported in Fig. 3.

### Cosmetic results

**Fig. 4:**
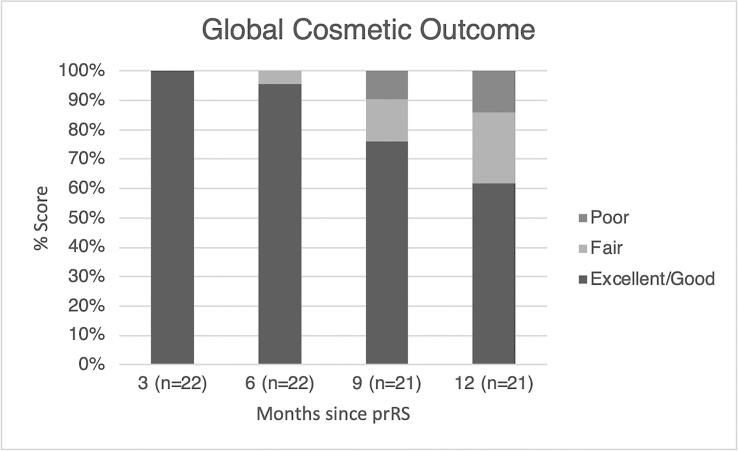
Physician’s cosmetic assessment over time.

Cosmetic outcomes worsened over time. After 6 months global cosmetic outcome was scored for 22 out of 22 patients; 21 (95.4 %) had a good to excellent and one (4.6 %) a fair cosmetic result. After 12 months (n = 21), the proportion of patients with a good to excellent cosmetic outcome was 76.2 % (n = 16), as compared to three fair (14.3 %) and two (9.5 %) poor outcomes. After 18 months global cosmetic outcome was available for 11 out of 22 patients; nine (81.8 %) had a good to excellent, one (9.1 %) a fair, and one (9.1 %) a poor cosmetic outcome. Of note, two out of three patients reporting poor cosmetic outcome reported over time had received previous contralateral breast surgery to treat benign breast disease. Physician’s cosmetic assessment over time is summarized in Fig. 4.

### Treatment efficacy

Overall, pCR after surgery according to the Chevalier score [24] was reported in two patients (9 %). Pathological positive axillary nodes (single sentinel lymph node biopsy with macrometastases receiving subsequent axillary lymph node dissection) were found in three out of 22 patients (13.6 %), and three out of 22 patients (13.6 %) had positive surgical margins (two patients reoperated). Postoperative whole breast irradiation was delivered, according to histopathological results, in two patients. Systemic adjuvant treatment was administered in 21 out 22 patients (95.4 %), 18 received exclusive endocrine treatment and three underwent adjuvant chemotherapy and endocrine treatment (Table 1). At time of analysis all patients reported no evidence of disease.

## Discussion

Preoperative breast irradiation represents a novel treatment strategy for early BC, with several potential advantages if compared to the current standard of care. It could potentially downstage larger breast cancers and improve cosmetic outcome [30]. The presence of the gross tumour also allows an increased accuracy in target definition during treatment planning, if compared to more challenging definition of a postoperative surgical bed [31]. However, it is not yet routine practice due to few disadvantages as compared to standard treatments: an increased need for an appropriate multidisciplinary evaluation of the patient starting from the early phases of diagnostic workflow; a potentially high interobserver variability in target contouring; the need for a careful assessment of diagnostic imaging (i.e., MRI) and clinical examination to warrant target delineation reliability [32]; a dedicated equipment requirement.

First results of the phase II ROCK trial showed that patients experienced low grade toxic effects after prRS using Cyberknife®, with no acute toxicity greater than G2. It allowed to warrant optimal non target tissue sparing, with mean heart and ipsilateral lung dose <1 Gy. Moreover, prRS did not delay both surgery and postoperative treatments, since no postsurgical complications were observed (i.e., wound dehiscence, infection, or skin necrosis). These data are consistent with a phase I dose escalation trial conducted by Horton and colleagues [18], testing a single-dose preoperative RT for unifocal early BC. Distinct dose-escalation levels of 15 Gy, 18 Gy, and 21 Gy were used. No acute dose-limiting radiation-related G3 toxicities or wound healing complications were observed, and no evidence of tumour progression was found at a median follow-up of 23 months. Thus, a 21 Gy single fraction treatment could be considered feasible and safe in a preoperative setting.

Concerning clinical outcomes, we observed two pCR after surgery (9 %). This relatively low rate of pCR might be underestimated, due to the short time interval between prRS and surgery. Patients included in the ROCK trial were affected by good-prognosis luminal-like HER2- disease, characterized by overall slow response kinetics. Considering that surgery was feasible in all the enrolled patients without any delay or complication, an adequate timing to ensure higher response to SBRT could be hypothesized to maximize benefit of pRS. Of course, such treatment approach should be considered with caution in patients affected by more aggressive biology disease. Bosma et al [16], discussing translational results from PAPBI trial to identify differences in gene expression between patients with and without response to RT, reported a 10 % rate of complete or near-complete pCR. Bondiau and colleagues [33], reported higher pCR rates in a single institution dose finding phase I study in locally advanced BC patients receiving neoadjuvant chemotherapy and preoperative robotic SBRT delivered as a boost. SBRT was delivered in three fractions on consecutive days using different dose-escalation levels: 19.5 Gy, 22.5 Gy, 25.5 Gy, 28.5 Gy, and 31.5 Gy. Surgery was performed 6–8 weeks after the last chemotherapy cycle followed by postoperative RT. Two patients experienced non-dose-limiting G2 toxicity, and one G3 skin dose-limiting toxicity was reported at dose level-4. A pCR was reported in nine out of the 25 patients (36 %). This improved pCR rate could have been related to the different time interval between RT and surgery, although response in patients affected by aggressive disease could be driven by systemic rather than local treatment. Biological predictive factors of response to RT in this scenario are unknown; translational research could allow to select patients likely to develop pCR, with a potential clinical benefit if compared to standard treatment [30].

The ABLATIVE single-arm prospective study assessed the pCR rate (primary endpoint) in patients with low-risk breast cancer treated with MRI guided preoperative PBI and to evaluated toxicity and patient-reported outcomes. Prescribed doses to GTV and CTV (CTV plus 20 mm margin) were 20 Gy and 15 Gy, respectively [34]. In the study, 36 patients were treated with a single ablative dose followed by BCS after an interval of 6 to 8 months, and pCR was reported in 15 patients (42 %) [34].

The differences in the numbers of pre-irradiation tumour infiltrating lymphocytes (TILs) between responders and non-responders after preoperative PBI in low-risk patients with breast cancer was also evaluated (22 pairs of pre-irradiation and post-irradiation tissue available). After preoperative PBI in this limited cohort, the number of TILs in tumour tissue decreased, although no differences in numbers of pre-irradiation TILs between responders and non-responders were observed [20].

Pathological positive axillary nodes were found in 13.6 % of patients, thus highlighting the importance of an accurate axillary imaging staging. No recurrences were detected in the biopsy track in patients enrolled within the ROCK trial. Of note, dissemination on biopsy track was detected within the PAPBI trial, prompting authors to suggest removal of the needle biopsy track to avoid this risk [17]. Smaller treatment volumes, associated with the high gradient offered by Cyberknife® robotic system, allow the use of an extreme hypofractionated schedule in this setting, with shorter treatment time as compared to the current conventional postoperative approach (i.e., 5 to 15 fractions). Thus, preoperative SBRT could be helpful to reduce socio-economic impact of radiation treatment and to increase patients’ compliance and health-related quality of life. Moreover, the identification of a subgroup of patients with a higher rate of pCR at time surgery, in whom an incremental clinical benefit may be detected and organ-preservation approach with surgery avoidance or treatment de-intensification could be further investigated within future clinical trials [9]. Conversely, main limitations of this approach might be related to the need for a highly complex comprehensive collaboration between all the involved breast specialists. Commenting the quite high drop-out rate as compared to the overall cohort of screened patients, selection criteria and multidisciplinary discussion represented a critical issue in this scenario. Moreover, pathologically positive axillary nodes were found in three patients, highlighting the importance of increasing sensitivity of preoperative nodal staging.

PAPBI was a phase II trial testing outcomes after a preoperative PBI regimen, published in 2020 [17]. Overall, 133 patients underwent an accelerated schedule consisting in 40 Gy in 10 fractions or 30 Gy in 5 fractions to the GTV, with a 25 mm expansion to obtain CTV. As compared to the ROCK trial, this study reported higher rate of postoperative complications (14 %) and a 10 % rate of 2-year moderate or greater fibrosis. Cosmetic outcomes were scored excellent-good in 68 % of patients at 6-month, and only four local recurrences were detected (three in the biopsy track and one in the ipsilateral breast). G1 acute skin toxicity was recorded in 34 % of cases, data in line with current results from ROCK trial.

In the ABLATIVE study, at a median follow-up of 21 months, all patients experienced grade 1 fibrosis in the treated breast volume. Transient grade 2 and 3 toxicity was observed in 31 % and 3 % of patients, respectively. Local recurrences were absent. No deterioration in patient reported outcomes or cosmetic results was observed [34].

A comparison between ROCK and PAPBI phase II trials in terms of efficacy and effectiveness was challenging due to the different length of follow up and the small sample size of our study. Conversely, a comparable cosmetic assessment was performed within the two trials [23], allowing a reliable comparison. Patients enrolled within the PAPBI trial experienced better overall cosmetic result, potentially related to the distinct treatment schedules used (single fraction *vs* hypofractionated regimen). Patients enrolled within ROCK trial underwent a treatment schedule corresponding to a BED_4_ of 131 Gy in all patients, while PAPBI trial provided two different treatment schedules corresponding to a BED_4_ of 140 Gy or 67.5 Gy in 59 % and 41 % of included patients, respectively. While the PAPBI reported an overall improvement over time, we observed a slight worsening of cosmetic outcome within the first year since prRS. To note, two patients with impaired cosmetic outcomes in our series have received previous contralateral surgery, with a potential non-negligible impact on breast symmetry before prRS.

Overall, the cosmetic outcome reported in this study seems to be worse than in several postoperative PBI studies [5], [6], [7], [8]: at 12 months, cosmetic outcome was good-excellent in only 60 % of patients (BCCT.core score), and is deteriorating in the first year, whilst the postoperative PBI trials of the same group showed the vast majority of patient reported cosmetic outcome scored as good to excellent. The ABLATIVE study also showed good to excellent results in >95 % of patients one year after a single 20 Gy fraction to the PTV [34]; the optimal preoperative single-fraction dose therefore remains an open question.

Interestingly 14 % of patients enrolled in PAPBI trial had postoperative complications, compared to none in our experience. Different timing of surgery (six weeks in PAPBI trial *vs* two weeks in our study) might also be responsible for this impairment, due to potential influence on RT-related connective tissue remodelling and inflammatory infiltrate impact on postoperative wound healing. Again, optimal timing for surgery is challenging, and future clinical trials should be aimed to find the correct workflow to maximise the therapeutic ratio.

## Conclusions

ROCK trial showed that a single 21 Gy dose prRS represents a feasible technique for selected patients affected by early BC, showing an acceptable preliminary toxicity profile. Our results encourage further investigations on this appealing treatment approach in larger studies, investigating prospective comparison with standard postoperative irradiation and translational biology-driven research studies.

## Declaration of Competing Interest

The authors declare the following financial interests/personal relationships which may be considered as potential competing interests: Icro Meattini reports occasional speaker honoraria supported by Eli Lilly, Novartis, Pfizer, Accuray, and Seagen, outside the submitted work. No other competing interests declared.
