# Supplementary material for: Preoperative robotic radiosurgery for early breast cancer: Results of the phase II ROCK trial (NCT03520894)
Source: Clin Transl Radiat Oncol. 2022 Sep 22;37:94–100. doi: 10.1016/j.ctro.2022.09.004 (PMC9513617; doi:10.1016/j.ctro.2022.09.004)
Supplement: Supplementary data 1 [file mmc1.docx]

**Supplementary Table 1.** Principal organs at risk dose constraints.

| **Ipsilateral breast** | V_10.5Gy_ <60%  V_22Gy_ <35% |
| --- | --- |
| **Contralateral breast** | <1Gy |
| **Ipsilateral lung** | V_7Gy_ <1000 cc |
| **Contralateral lung** | <1Gy |
| **Heart** | V_3Gy_ <5 cc |
| **Skin** | V_10Gy_ <10 cc  V_20Gy_ <1 cc |
| **Chest wall** | V_10Gy_ <10 cc |
