# Supplementary material for: Preoperative robotic radiosurgery for early breast cancer: Results of the phase II ROCK trial (NCT03520894)
Source: Clin Transl Radiat Oncol. 2022 Sep 22;37:94–100. doi: 10.1016/j.ctro.2022.09.004 (PMC9513617; doi:10.1016/j.ctro.2022.09.004)
Supplement: Supplementary data 2 [file mmc2.docx]

**Supplementary Table 2.** Translational research module methods.

| **Rationale** |
| --- |
| *1. Immune infiltrate analysis* |
| Cancer grows in dynamic equilibrium with the immune system. The capacity of tumour cells to modify their microenvironment is an essential component in tumour progression, and inflammation plays an important role in this procession. Irradiation alters the equilibrium by modifying the immune tumour microenvironment. Ionizing radiations modulate pre-existing immune cells and facilitate leukocyte infiltration by induction of chemotactic signals and by upregulation of adhesion molecule expression by the tumour endothelium [1]. The impact of irradiation, and probably the outcome of treatment, therefore, depends on the pre-existing status quo and on the quality and quantity of leukocyte in the tumour microenvironment [2, 3]. Growing evidence support that tumour associate macrophages (TAM) M1 or M2 phenotype and tumour infiltrating lymphocytes (TILs) are crucial in determining the prognosis and outcome of patients at any clinical stage [4]. For this reason, we planned a qualitative and a quantitative analysis of macrophage and lymphocyte subpopulations, both in pre-irradiation biopsies and postoperative tumour specimens. Moreover, we related the results to radiation response and to outcome. |
| *2. Selected gene expression analysis* |
| Many strides have been made in the comprehension of breast cancer biology. Early-stage breast cancer is a well-recognized disease for its clinical and genomic heterogeneity. Hormone receptor status, HER2 status, Ki 67 index and tumour size and extent of nodal involvement, represent the traditional prognostic factors of early-stage breast cancer. However, in the era of personalized treatments, these factors appeared insufficient for optimum decision making [5, 6]. Since standard clinical pathological parameters still drive adjuvant RT, a fully personalized RT has yet to be introduced in the clinical setting. Moreover, benefits of RT are not equal for all patients with breast cancer and across risk groups. To improve cure and/or reduce toxicity, the identification of prognostic and/or predictive factors still represents the main challenge to facilitate decision-making in whether to administer RT and escalate or de-escalate dosages and volumes. The identification of gene expression signatures can allow to evaluate the risk of recurrence for women with early breast cancer and helps to individualize the treatment decisions. A genomic signatures, in attempts to decipher the tumour’s genetic fingerprint and associated risk of local or loco-regional relapse, may represent a potential predictive factor and leads to identify patients most likely to benefit from a given treatment [7]. For this reason, we decided to analyse a pool of genes associated with breast cancer and/ or radiotherapy which may have the potential to become predictive factor and help in the definition of a personalized treatment. Our analyses base on the evaluation of gene expression and potential correlation to outcome and radiotherapy response. |
| **Material and methods** |
| *1. Clinical specimens* |
| Fresh biopsies and fresh postoperative tumour material were retrieved from 22 patients. Patients selected for this study gave an informed consent. |
| *2. Histology, immunohistochemistry (IHC)* |
| Biopsies specimens and postoperative tumours were dissected and fixed in 4% PFA. For IHC, sections were stained with CD4 and CD8 (mouse; Santa cruz biotechnology inc, 1:200- 1:300) for lymphocytes, with CD11b, CD14, CD68, CD86, CD206 (mouse; Santa cruz biotechnology inc, 1:200- 1:300), CD163 (mouse, Novus biologicals,1:200) for TAM evaluation, and with CD34 (mouse; Santa cruz biotechnology inc, 1:200) for vascular architecture. IHC was performed using the Leica BOND-MAX™ automated system (Leica Microsystems). Slides were developed with 3′3-diaminobenzidine (Leica Microsystems) and counterstained with haematoxylin. The specificity of the antibodies was established using a negative and a positive human-tissue sample. Images were acquired by using a slide scanner (Aperio LV1; Leica Biosystems) and analysed with the software ImageScope For pathological assessment and evaluation of necrosis 4-μm lungs sections were cut and stained using haematoxylin and eosin (HE). Pathological assessment was calculated by the Chevallier grade that considers the presence of some regression and pathologic response was classified as follows: Grade 1(disappearance of all tumour on both macroscopic and microscopic examination); Grade 2 (presence of in situ carcinoma of the breast, no invasive tumour, and no tumour found in lymph nodes); Grade (presence of invasive carcinoma with stromal alterations, such as sclerosis or fibrosis); Grade 4 (no or few alterations in tumour appearance). Necrosis was evaluated by pathology specialists as present or absent. |
| *3. RNA isolation, Taqman array cards and qRT-PCR analyses* |
| Total RNAs were extracted from frozen samples using mirVana miRNA isolation kit (Ambion) according to the manufacturer’s instructions. The integrity and quantity of RNAs were assessed by spectrophotometry (NanoDrop Technologies). For gene analysis, quantitative RT-PCR was performed for each sample using TaqMan Array microfluidic cards (Applied Biosystems, Foster City, CA) and ABI 7500 Real Time PCR System (Applied Biosystems). All assays were performed in triplicates. |
| **References** |
| [1] Burnette B, Weichselbaum RR. Radiation as an immune modulator. Semin Radiat Oncol. 2013;23:273-80.  [2] Dieci MV, Radosevic-Robin N, Fineberg S, van den Eynden G, Ternes N, Penault-Llorca F, et al. Update on tumor-infiltrating lymphocytes (TILs) in breast cancer, including recommendations to assess TILs in residual disease after neoadjuvant therapy and in carcinoma in situ: A report of the International Immuno-Oncology Biomarker Working Group on Breast Cancer. Semin Cancer Biol. 2018;52:16-25.  [3] Demaria S, Formenti SC. Radiation as an immunological adjuvant: current evidence on dose and fractionation. Front Oncol. 2012;2:153.  [4] Demaria S, Formenti SC. Role of T lymphocytes in tumor response to radiotherapy. Front Oncol. 2012;2:95.  [5] Liu MC, Pitcher BN, Mardis ER, Davies SR, Friedman PN, Snider JE, et al. PAM50 gene signatures and breast cancer prognosis with adjuvant anthracycline- and taxane-based chemotherapy: correlative analysis of C9741 (Alliance). NPJ Breast Cancer. 2016;2.  [6] Varnier R, Sajous C, de Talhouet S, Smentek C, Peron J, You B, et al. Using Breast Cancer Gene Expression Signatures in Clinical Practice: Unsolved Issues, Ongoing Trials and Future Perspectives. Cancers (Basel). 2021;13.  [7] Aristei C, Perrucci E, Ali E, Marazzi F, Masiello V, Saldi S, et al. Personalization in Modern Radiation Oncology: Methods, Results and Pitfalls. Personalized Interventions and Breast Cancer. Front Oncol. 2021;11:616042. |
